# MVA-CoV2-S Vaccine Candidate Neutralizes Distinct Variants of Concern and Protects Against SARS-CoV-2 Infection in Hamsters

**DOI:** 10.3389/fimmu.2022.845969

**Published:** 2022-03-16

**Authors:** Robbert Boudewijns, Patricia Pérez, Adrián Lázaro-Frías, Dominique Van Looveren, Thomas Vercruysse, Hendrik Jan Thibaut, Birgit Weynand, Lotte Coelmont, Johan Neyts, David Astorgano, Dolores Montenegro, Eugenia Puentes, Esteban Rodríguez, Kai Dallmeier, Mariano Esteban, Juan García-Arriaza

**Affiliations:** ^1^ KU Leuven Department of Microbiology, Immunology and Transplantation, Rega Institute, Laboratory of Virology and Chemotherapy, Leuven, Belgium; ^2^ Department of Molecular and Cellular Biology, Centro Nacional de Biotecnología (CNB), Consejo Superior de Investigaciones Científicas (CSIC), Madrid, Spain; ^3^ Centro de Investigación Biomédica en Red de Enfermedades Infecciosas (CIBERINFEC), Madrid, Spain; ^4^ KU Leuven Department of Microbiology, Immunology and Transplantation, Rega Institute, Translational Platform Virology and Chemotherapy (TPVC), Leuven, Belgium; ^5^ KU Leuven Department of Imaging and Pathology, Translational Cell and Tissue Research, Leuven, Belgium; ^6^ Biofabri, Pontevedra, Spain

**Keywords:** SARS-CoV-2, COVID-19, MVA vaccine, spike, hamsters, immunogenicity, efficacy

## Abstract

To control the coronavirus disease 2019 (COVID-19) pandemic and the emergence of different variants of concern (VoCs), novel vaccines against severe acute respiratory syndrome coronavirus 2 (SARS-CoV-2) are needed. In this study, we report the potent immunogenicity and efficacy induced in hamsters by a vaccine candidate based on a modified vaccinia virus Ankara (MVA) vector expressing a human codon optimized full-length SARS-CoV-2 spike (S) protein (MVA-S). Immunization with one or two doses of MVA-S elicited high titers of S- and receptor-binding domain (RBD)-binding IgG antibodies and neutralizing antibodies against parental SARS-CoV-2 and VoC alpha, beta, gamma, delta, and omicron. After SARS-CoV-2 challenge, MVA-S-vaccinated hamsters showed a significantly strong reduction of viral RNA and infectious virus in the lungs compared to the MVA-WT control group. Moreover, a marked reduction in lung histopathology was also observed in MVA-S-vaccinated hamsters. These results favor the use of MVA-S as a potential vaccine candidate for SARS-CoV-2 in clinical trials.

## Introduction

The severe acute respiratory syndrome coronavirus 2 (SARS-CoV-2) emerged at the end of 2019, causing the coronavirus disease 2019 (COVID-19), a condition that can lead to life-threatening pneumonia (1), which resulted in a pandemic that is producing an important global healthcare crisis. Currently, the pandemic is driven by variants of concern (VoCs) that have emerged from the parental Wuhan SARS-CoV-2 strain (B lineage and early offspring thereof, such as B.1 strain), such as alpha (B.1.1.7), beta (B.1.351), gamma (P.1), delta (B.1.617.2), and most recently omicron (B.1.1.529) (2, 3). Given the persistently high burden of SARS-CoV-2 since its emergence, the world has pinned its hopes on the development of effective vaccines and therapeutics to curb the ongoing pandemic. At the time of writing, eight COVID-19 vaccines have been listed for use by the World Health Organization and regulatory agencies, and large-scale vaccination campaigns are well underway in many countries. A common feature of these vaccines is their presentation of some form of the SARS-CoV-2 spike (S) glycoprotein, the main target for neutralizing antibodies (nAbs) (4). The S protein mediates viral entry through interaction of its receptor-binding domain (RBD) with the host receptor angiotensin-converting enzyme 2 (ACE2) (5, 6).

Despite the existence of COVID-19 vaccines approved for use in humans, and many more currently in clinical development, the nature of the immune response that is required for protection against virus infection and disease is still not completely understood. Since recent studies have demonstrated that vaccine regimens consisting of a combination of vaccines elicit strong immune responses (7), additional novel vaccine approaches may offer unique contributions to protective immunity and cover shortcomings of individual vaccines when used in combination. Moreover, as currently available vaccines comprise a sequence of the S protein from the ancestral B strain, it remains unclear how well they will perform against novel SARS-CoV-2 VoCs and new variants that may emerge in the future. Additionally, with vaccine shortages in the developing world, and the administration of a third dose to boost anti-SARS-CoV-2 immunity, more vaccine candidates are needed to increase the likelihood of meeting the global demand for COVID-19 control.

Among the different vaccine approaches, one of the most promising is the use of viral vectors, such as poxviruses, which have shown potent immunogenicity and efficacy in preclinical and clinical trials against several infectious diseases (8, 9). In fact, we have previously reported the high immunogenicity and efficacy profile in vaccinated mice of a COVID-19 vaccine candidate based on the highly attenuated modified vaccinia virus Ankara (MVA) vector expressing the full-length SARS-CoV-2 S protein, termed MVA-S (10–12). MVA-S induced robust SARS-CoV-2-specific T-cell and humoral immune responses in C57BL/6 mice, and one or two doses of MVA-S fully protected susceptible K18-hACE2 transgenic mice against lethal SARS-CoV-2 challenge (10–12).

It has been previously reported that Syrian hamsters are naturally susceptible to SARS-CoV-2 infection (13–15). Intranasal inoculation of hamsters with SARS-CoV-2 results in viral replication in the upper and lower respiratory tract (15) and an inflammation of the lungs hallmarked by leukocyte infiltration, edema, and overexpression of inflammatory cytokines (13, 15), similar to human COVID-19. In the current study, we showed that administration of one or two doses of MVA-S in Syrian hamsters resulted in the potent induction of S- and RBD-specific IgG antibodies that neutralized SARS-CoV-2 VoCs alpha, beta, gamma, delta, and omicron. Remarkably, MVA-S vaccination triggered reduced levels of SARS-CoV-2 RNA and infectious virus in the lungs of hamsters infected with SARS-CoV-2 and protected all hamsters against SARS-CoV-2-induced lung disease as seen by histopathological scoring. These results warrant the further development of MVA-S toward clinical trials.

## Materials and Methods

### Animals and Ethics Statement

Female Syrian hamsters (*Mesocricetus auratus*) that were 6–8 weeks old were sourced from Janvier Laboratories (Le Genest-Saint-Isle, France). Hamsters were housed in pairs in individually ventilated cages (GR900 Sealsafe Plus, Tecniplast, Buguggiate, Italy) with food and water *ad libitum*, at 21°C, 55% humidity, and 12:12 dark/light cycles. Extra bedding material and wooden gnawing blocks were provided as cage enrichment. The ethical committee of KU Leuven (Belgium) approved housing conditions and experimental procedures (license p015/2020) according to institutional guidelines approved by the Federation of European Laboratory Animal Science Associations (FELASA). Animals were monitored for signs of disease (lethargy, heavy breathing, ruffled fur, hunched posture, and agitation) and weight loss during the course of the study.

### Cells

Vero E6 cells (KU Leuven: kind gift from Peter Bredenbeek, LUMC, Netherlands; CNB-CSIC: ATCC CRL-1586) were maintained in Minimum Essential Medium (MEM; Gibco-Life Technologies) supplemented with 10% fetal bovine serum (FBS; Hyclone), 1% L-glutamine (Gibco-Life Technologies) and 1% sodium bicarbonate (Gibco-Life Technologies) (at KU Leuven) or in Dulbecco’s modified Eagle’s medium (DMEM) supplemented with 10 mM HEPES (4-(2-hydroxyethyl)-1-piperazineethanesulfonic acid) (Gibco-Life Technologies), 1X nonessential amino acids (Gibco-Life Technologies), penicillin (100 U/ml, Sigma-Aldrich), streptomycin (100 mg/ml, Sigma-Aldrich), and 10% heat-inactivated FBS (Gibco-Life Technologies) (at CNB-CSIC). All assays were performed in medium containing 2% FBS instead of 10%. Cell cultures were maintained at 37°C in a humidified incubator containing 5% CO_2_. HEK293T (human embryonic kidney, ATCC CRL-3216) cells were maintained in DMEM (Gibco-Life Technologies), supplemented with 10% FBS (Hyclone), 2 mM L-glutamine (Gibco-Life Technologies), and 1% sodium bicarbonate (Gibco-Life Technologies).

### MVA-S Vaccine

MVA-S vaccine candidate expresses a human codon optimized full-length SARS-CoV-2 S protein (strain B.1), and its generation was previously described (10). MVA-S vaccine candidate was manufactured according to current Good Manufacturing Practice by the company Biofabri (Spain). MVA-S virus was grown in cultured chicken cells (DF-1), harvested, clarified and purified by Tangential Flow Filtration, vialed, and stored at -15°C to -30°C. MVA-WT virus is an attenuated poxvirus strain, obtained from the Chorioallantois vaccinia virus Ankara strain after 586 serial passages in CEF cells (16), and was grown in DF-1 cells and purified by centrifugation through two 36% (wt/vol) sucrose cushions in 10 mM Tris-HCl (pH 9). MVA virus titers were determined by immunostaining, as previously described (17).

### SARS-CoV-2 Virus

SARS-CoV-2 virus stock derived from prototypic strain B.1 (BetaCov/Belgium/GHB-03021/2020, EPI_ISL_407976|2020-02-03), VoC B.1.1.7 (alpha; hCoV-19/Belgium/rega-12211513/2020; EPI_ISL_791333, 2020-12-21), B.1.351 (beta; hCoV-19/Belgium/rega-1920/2021; EPI_ISL_896474, 2021-01-11), and B.1.1.529 (omicron; hCoV-19/Belgium/rega-20174/2021, EPI_ISL_6794907) have been previously described (13, 18, 19). VoC P.1 (gamma; hCoV-19/Belgium/rega-3278/2021, EPI_ISL_1091366) and B.1.167.2 (delta; hCoV-19/Belgium/rega-7214/2021, EPI_ISL_2425097) were isolated from a Belgian patient and characterized by next-generation sequencing. SARS-CoV-2 MAD6 isolate is similar to the B.1 strain but contains the D614G mutation in the S protein and has been previously described (10, 20).

All SARS-CoV-2 virus stocks were grown on Vero E6 cells for two (B.1.1.7, B.1.351, P.1, B.1.167.2, B.1.1.529, MAD6) or three (B.1) passages. Virus stocks were free from mycoplasma (PlasmoTest, InvivoGen), and deep sequencing on a MiSeq platform (Illumina) confirmed that stocks contained no other adventitious agents. Infectious virus content was determined after titration on Vero E6 cells by the method of Spearman–Kärber and expressed as median tissue culture infectious dose (TCID_50_). All virus-related work was conducted in the BSL-3 facilities of the KU Leuven Rega Institute (Belgium) or at the CNB-CSIC (Spain), according to institutional guidelines.

### Study Schedule, Vaccinations, and SARS-CoV-2 Challenge

A total of 36 animals were used, divided into three groups (n = 12/group). On day 0, group 1 received 2 × 10^7^ plaque-forming units (PFU) of MVA-S, group 2 received PBS, and group 3 received 2 × 10^7^ PFU of MVA-WT. On day 21, groups 1 and 2 received 4 × 10^7^ PFU of MVA-S, and group 3 received 4 × 10^7^ PFU of MVA-WT. All vaccinations were performed by intraperitoneal (i.p.) injection in a final volume of 100 µl. At days -3, 21, and 39, all animals were bled from the jugular vein under isoflurane anesthesia. On day 42, all animals were infected intranasally (i.n.) with 2 × 10^5^ TCID_50_ of SARS-CoV-2 (B.1) in 50 µl culture medium (MEM, 2% FBS) under isoflurane anaesthesia. On each sampling day after SARS-CoV-2 infection [2, 4, and 14 days post-infection (dpi)], four hamsters of each group were euthanized by i.p. injection of 500 µl Dolethal (200 mg/ml sodium pentobarbital, Vétoquinol SA, Aartselaar, Belgium), and lung tissue was collected in 4% formalin, MEM, or lysis buffer (E.Z.N.A. Total RNA Kit I, Omega Bio-Tek) for histopathological analysis, virus titration, and RT-qPCR, respectively. All manipulations were performed under a laminar flow cabinet.

### Enzyme-Linked Immunosorbent Assay

Individual serum samples obtained from hamsters at day 39 were tested for the presence of binding IgG antibodies against SARS-CoV-2 S and RBD proteins using an ELISA, as previously described (10). The S and RBD proteins used to coat the plates were derived from the Wuhan-Hu-1 strain (GenBank accession number MN908947.3) and were previously described (10). In the S protein (residues 1 to 1,208), the furin-recognition motif Arg-Arg-Ala-Arg was replaced by the Gly-Ser-Ala-Ser sequence, and it also contained the Ala942Pro, Lys986Pro, and Val987Pro substitutions in the S2 portion. The RBD protein spanned residues 332 to 534 of the S protein. Total binding IgG titers were measured as the last serum dilution that gives an absorbance value at 450 nm at least three times higher the absorbance of serum from day -3 (pre-immune serum).

### Indirect Immunofluorescence Assay

SARS-CoV-2-specific binding antibodies at days 21 and 39 were also determined by indirect immunofluorescence assay (IIFA), as previously described (21). Briefly, serial dilutions of serum were made on wild-type or SARS-CoV-2 S-expressing HEK293T cells. Cells were subsequently stained with goat-anti-mouse IgG Alexa Fluor 488 (A11001, Life Technologies; 1:250 dilution) and 4′,6-diamidino-2-phenylindole (DAPI). Finally, IIFA titers were determined as the highest serum dilution from which a positive signal could be obtained.

### SARS-CoV-2 Neutralization

Live-virus SARS-CoV-2 nAbs were measured at day 39 using a microneutralization test (MNT) assay in a BSL-3 laboratory at the CNB-CSIC. Serially 2-fold diluted serum samples in DMEM-2% FBS medium were incubated at a 1:1 ratio with 100 TCID_50_ of SARS-CoV-2 MAD6 isolate (having the D614G mutation in the S protein) in 96-well tissue culture plates for 1 h at 37°C. Then, mixtures of serum samples and SARS-CoV-2 virus were added in duplicate to Vero-E6 cell monolayers seeded in 96-well plates at 30,000 cells/well, and plates were incubated at 37°C in a 5% CO_2_ incubator for 3 days. Then, cells were fixed with 10% formaldehyde for 1 h and stained with crystal violet. When plates were dried, crystal violet was diluted in H_2_O-10% sodium dodecyl sulfate (SDS), and optical density was measured in a luminometer at 570 nm. Neutralizing titer 50 (NT_50_) was calculated as the reciprocal dilution resulting in 50% inhibition of cell death following a methodology previously described (22).

Additionally, nAb titers against SARS-CoV-2 (B.1) were quantified at days 21 and 39 in an in-house-developed serum neutralization test (SNT) with green fluorescent protein (GFP)-expressing vesicular stomatitis virus (VSV) pseudotypes carrying the SARS-CoV-2 (B.1) S, as previously described (21). In brief, serial serum dilutions were incubated at 37°C for 1 h with an equal volume of S-pseudotyped VSV particles and subsequently inoculated on Vero E6 cells for 18 h. Median NT_50_ neutralization titers were determined by non-linear regression curve fitting of the percentages of green cells measured by a high-content imager (Cell Insight CX5 High Content Screening platform, Thermo Fisher Scientific).

### Neutralization of SARS-CoV-2 Variants of Concern

Serum obtained at day 39 from hamsters vaccinated twice with MVA-S was pooled and tested for neutralization against SARS-CoV-2 VoC using a cytopathic effect (CPE)-based neutralization test (CPENT), as previously described (23). In brief, serial 2-fold dilutions (1:4, 1:8, 1:16, 1:32, 1:64, and 1:128) of pooled serum were incubated for 1 h at 37°C with equal volumes of SARS-CoV-2 virus containing 100 TCID_50_ (prototypic strain B.1 and VoC B.1.1.7, B.1.351, P.1, B.1.167.2, and B.1.1.529). This mixture was then put onto a Vero E6 cell layer (2 × 10^4^ cells/well in 96-well plates) and incubated for 3 days at 37°C, after which the percentage of CPE was scored visually. Cell survival in each well relative to the mean of the virus control (VC) was calculated as follows: % live cells = (%CPE_well_-%CPE_VCmean_)/(%CPE_CellControlmean_-%CPE_VCmean_) × 100. The median inhibitory concentration (IC_50_) of the serum was then obtained by non-linear curve fitting on the percentage of live cells as a function of the serum concentration. All assays were performed in triplicate.

### RNA Extraction and Quantification of SARS-CoV-2 Subgenomic RNA by RT-qPCR

RNA was extracted from 30 mg of homogenized lung tissue using the E.Z.N.A. Total RNA Kit I (Omega Bio-Tek), following the manufacturer’s instructions. SARS-CoV-2 subgenomic RNA copies (N gene) were quantified by RT-qPCR, as previously described (13). The relative fold change of SARS-CoV-2 subgenomic RNA levels was calculated by the 2^-ΔΔCq^ method with β-actin RNA levels for normalization.

### Infectious Virus Titration

For quantification of infectious SARS-CoV-2 viral particles, the supernatant of homogenized and centrifuged lung tissue was incubated on confluent Vero E6 cells. Infectious viral titers were calculated after 3 days by the method of Spearman and Kärber (24) and expressed as TCID_50_ per 100 mg of homogenized lung tissue.

### Lung Histopathology

Lungs were fixed in 4% paraformaldehyde in PBS for a minimum of 24 h and then embedded in paraffin. Tissue sections of 4 µm were stained with hematoxylin and eosin and scored for signs of lung damage by an expert pathologist, as previously described (18). Scores of 0–3 were given for the following parameters: intra-alveolar edema, lymphoid follicles, apoptotic bodies in the bronchi walls, necrotizing bronchiolitis, perivascular edema, bronchopneumonia, perivascular cuffing, peribronchiolar inflammation, vasculitis, and perivascular inflammation.

### Statistical Analysis

All statistical evaluations were performed with GraphPad Prism Version 9.1.2 (GraphPad Software, Inc.). Data are presented as means ± SEM. Statistical significance between conditions was calculated using the non-parametric Mann–Whitney test (significance at p-values <0.05).

## Results

### MVA-S Vaccination in Hamsters Elicited S-Specific Antibodies That Neutralized SARS-CoV-2 Variants of Concern

**Figure 1:**
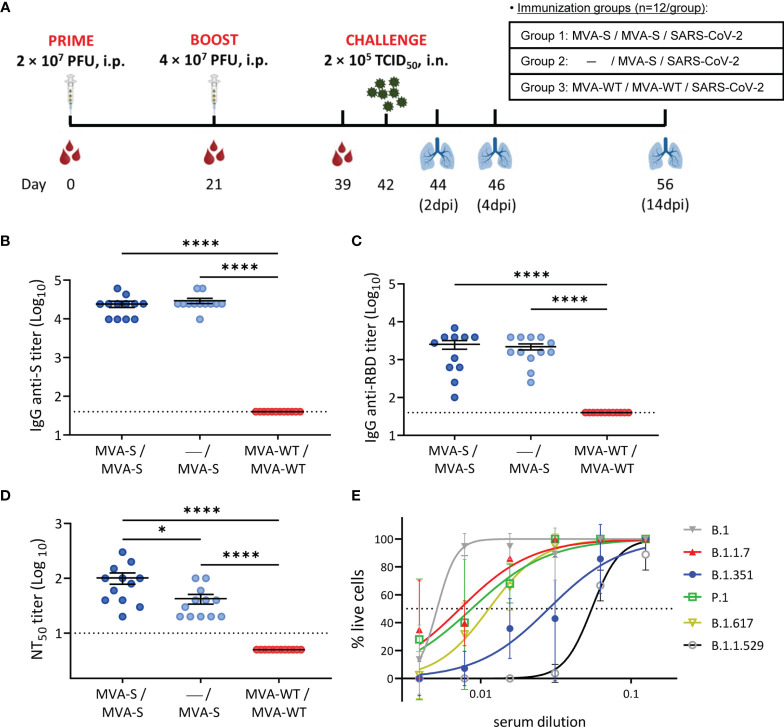
Immunization schedule and analysis of SARS-CoV-2-specific humoral immune responses induced by MVA-S vaccination in hamsters. **(A)** Experiment overview. Syrian hamsters (n = 12 per group) were immunized by the intraperitoneal (i.p.) route with two doses of MVA-S or MVA-WT at days 0 and 21 or one dose of MVA-S at day 21 and challenged intranasally (i.n.) with 2 × 10^5^ TCID_50_ (median tissue culture infectious dose) of SARS-CoV-2 B.1 strain at day 42, as indicated. Blood was collected before boosting (day 21 post prime immunization) and before challenging (day 39, 18 days post-boost). On 2, 4, and 14 days post-infection (dpi), 4 animals per group were sacrificed and their lungs were collected for virological and histological analysis. **(B, C)** Titers of anti-S **(B)** and anti-receptor-binding domain (RBD) **(C)** binding IgG antibodies determined by ELISA in serum collected on day 39. Mean values and SEM are represented. Dashed line represents the limit of detection. **(D)** NT^50^ (50% neutralization) titers were evaluated in serum collected on day 39 using a live virus microneutralization assay with SARS-CoV-2 MAD6 isolate. Mean NT^50^ values ± standard error of the mean (SEM) are represented. **(E)** SARS-CoV-2 neutralizing antibody titers against SARS-CoV-2 VoC. Pooled serum from hamsters vaccinated twice with MVA-S and obtained at day 39 was used in a cytopathic effect (CPE)-based neutralization assay against different SARS-CoV-2 variants of concern (VoCs). Median inhibitory concentrations (IC_50_, dotted line) per variant were calculated by non-linear curve fitting of the percentage of live cells. Data presented as means ± SEM. Statistical significance between groups was calculated by Mann–Whitney test (*p < 0.05, ****p < 0.0001).

To assess the ability of the MVA-S vaccine candidate to elicit SARS-CoV-2-specific binding and nAbs, groups of female Syrian hamsters (n = 12 per group) were immunized in a one- or two-dose regimen with MVA-S (**Figure 1A**). One group of animals received a prime dose of 2 × 10^7^ PFU of MVA-S *via* the i.p. route on day 0, followed by a booster dose with 4 × 10^7^ PFU of MVA-S at day 21 (MVA-S/MVA-S), whereas a second group only received a single dose of 4 × 10^7^ PFU of MVA-S at day 21 (—/MVA-S). A third group primed and boosted with similar doses of MVA-WT at days 0 and 21 served as the control group (MVA-WT/MVA-WT) (**Figure 1A**). Vaccination with MVA-S or MVA-WT had no effect on body weight progression (**Figure S1A**).

For serological analysis, serum was collected at days 21 (before boosting; 21 days post prime) and 39 (before SARS-CoV-2 challenge; 18 days post boost). First, we analyzed the presence of anti-S and anti-RBD-binding IgG antibodies at day 39 by ELISA. The results showed that single and double MVA-S vaccination elicited similarly high IgG titers against the S protein (**Figure 1B**) and more specifically against the RBD (**Figure 1C**). These results were also confirmed by an IIFA assay against the S protein, with induction of high titers of binding antibodies at 21 days post prime MVA-S immunization (**Figure S2A**) that were further enhanced after the booster dose (**Figure S2B**).

Next, the evaluation of the levels of SARS-CoV-2 nAbs at day 39 by using a live microneutralization assay showed that a single and double dose of MVA-S elicited high titers of nAbs against SARS-CoV-2 (MAD6 strain, containing D614G mutation in the S protein) (**Figure 1D**), with significantly higher titers in the two-dose MVA-S regimen compared to one dose of MVA-S and with no neutralizing activity observed in the MVA-WT control group. Additionally, a neutralization assay using S-pseudotyped VSV particles yielded similar results, with induction of high nAb titers already at 21 days after the first MVA-S dose (**Figure S2C**) that were further enhanced after the booster dose (**Figure S2D**), although in 2 out of 12 animals, no nAbs were detected with the S-VSV pseudovirus (**Figure S2D**).

To address the increasing clinical importance of SARS-CoV-2 VoC and neutralizing capacity of the MVA-S vaccine, pooled serum samples obtained at day 39 from double-vaccinated hamsters were tested for neutralization against SARS-CoV-2 VoC alpha (B.1.1.7), beta (B.1.351), gamma (P.1), delta (B.1.167.2), and omicron (B.1.1.529) and compared to the prototypic SARS-CoV-2 B.1 strain (**Figure 1E**). As expected, the median inhibitory concentration (IC_50_ ± SEM) of serum was the lowest for neutralization of the prototypic B.1 strain (5.14 ± 0.52 × 10^-3^), shortly followed by that of the alpha (7.30 ± 1.06 × 10^-3^), gamma (8.72 ± 1.95 × 10^-3^), and delta (1.14 ± 0.11 × 10^-2^) strains. The beta and omicron VoCs could also be neutralized, although at higher serum concentrations (2.83 ± 0.54 × 10^-2^ for beta and 5.48 ± 0.23 × 10^-2^ for omicron).

### MVA-S Vaccination Prevented SARS-CoV-2 Replication and Lung Pathology in Hamsters

**Figure 2:**
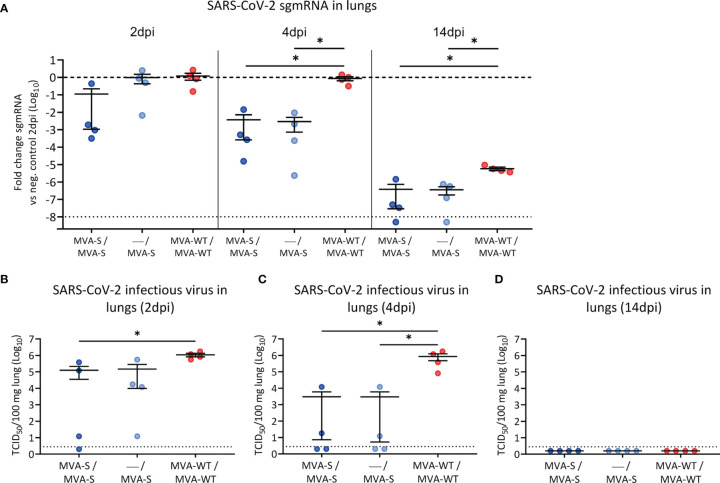
SARS-CoV-2 virus replication in lung samples. SARS-CoV-2 sgmRNA levels **(A)** and infectious viral loads **(B–D)** in lungs of vaccinated hamsters. **(A)** SARS-CoV-2 sgmRNA levels were normalized against levels of β-actin, and fold changes compared to the MVA-WT control group of day 2 post-infection were calculated using the 2^-ΔΔCq^ method. Dashed line indicates the mean of the control group. **(B–D)** Infectious viral loads in lungs of hamsters on day 2 **(B)**, day 4 **(C)**, and day 14 **(D)** post-infection are expressed as the number of infectious virus particles per 100 mg of lung tissue. Dotted line indicates the limit of detection. Data presented as means ± SEM. Statistical significance between groups was calculated by Mann–Whitney test (*p < 0.05).

Three weeks after the last vaccine dose (day 42), all hamsters were infected intranasally with 2 × 10^5^ TCID_50_ of SARS-CoV-2 (**Figure 1A**). Initially, body weight was analyzed after challenge, and hamsters from all groups experienced a similar slight drop in body weight after SARS-CoV-2 infection, but by day 4 post-challenge, vaccinated groups had significantly recovered compared to control animals (**Figure S1B**). At 2, 4, and 14 dpi, four animals per group were sacrificed, and their lungs were analyzed for signs of SARS-CoV-2 replication and virus-induced lung damage. MVA-S vaccination resulted in reduced levels of SARS-CoV-2 subgenomic (sgm) RNA (N gene) in double-vaccinated hamsters already at 2 dpi (**Figure 2A**). At 4 dpi, all MVA-S-vaccinated animals, even after a single dose, had approximately a significant 10^3^-fold reduction of SARS-CoV-2 sgmRNA levels in their lungs compared to the MVA-WT control group (**Figure 2A**). At 14 dpi, overall viral sgmRNA levels had gone down but were still significantly reduced in hamsters vaccinated with one or two doses of MVA-S (**Figure 2A**). Infectious virus titers were consistently reduced in lungs of vaccinated animals at 2 and 4 dpi (**Figures 2B, C**), and at 4 dpi, even up to a significant 10^5^-fold reduction, with 2 out of 4 animals in each vaccinated group having no detectable virus (**Figure 2C**). At 14 dpi, the infectious virus had disappeared from the lungs in all groups (**Figure 2D**).

**Figure 3:**
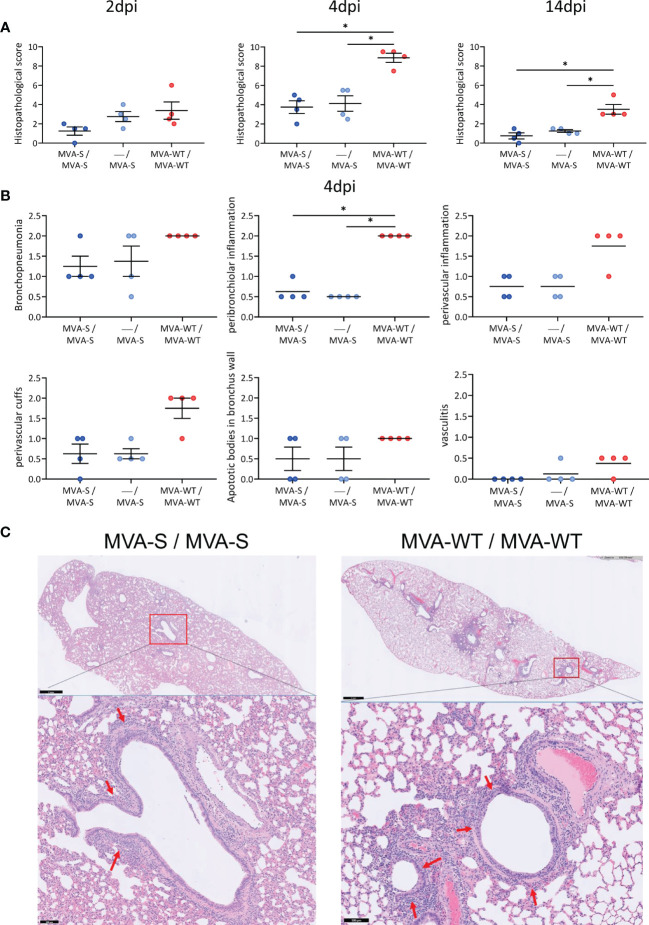
Lung histopathology. **(A)** Histopathological scores of hematoxylin and eosin-stained hamster lung sections on 2, 4, and 14 days post-infection (dpi). Data presented as means ± SEM. Statistical significance between groups was calculated by Mann–Whitney test (*p < 0.05). **(B)** Histopathological scores of day 4 post-challenge are plotted per histopathological sign. Statistical significance between groups was calculated by Mann–Whitney test (*p < 0.05). **(C)** Representative hematoxylin and eosin-stained images of lung sections from hamsters at day 4 after virus challenge. General view of the lung (upper) along with histopathological details from selected lung areas (red boxes) have been displayed (lower). Lung sections from vaccinated MVA-S/MVA-S and MVA-WT/MVA-WT control hamsters are represented. Red arrows (lower panel) indicate peribronchiolar inflammation. Scale bars: 1 mm (upper) and 100 µm (lower).

Resembling COVID-19 bronchopneumonia in humans, SARS-CoV-2 infection causes lung pathology in hamsters (13), which is hallmarked by inflammation, edema, and infiltration of leukocytes, and can be readily observed by histological staining on lung sections already a few days after infection. The lung pathology analysis showed that, compared to MVA-WT control animals, hamsters vaccinated with one or two doses of MVA-S had a significant reduction at 4 and 14 dpi in histopathological scores (**Figure 3A**). A detailed analysis at 4 dpi (the peak of lung histopathology in control animals) showed that animals vaccinated with one or two doses of MVA-S have lower scores of bronchopneumonia, peribronchiolar and perivascular inflammation, perivascular cuffs, apoptotic bodies in bronchus wall, and vasculitis compared to MVA-WT control animals (**Figure 3B**). Representative hematoxylin and eosin-stained images of lungs after virus challenge showed a clear inflammation around bronchi and vascular structures that extended into the surrounding tissue in MVA-WT control animals, even at low magnification, whereas in MVA-S-vaccinated animals, only limited inflammation could be observed (**Figure 3C**). Despite some hamsters having a clear lung pathology, no obvious signs of disease (lethargy, heavy breathing, ruffled fur, hunched posture, and agitation) could be seen.

## Discussion

Although several COVID-19 vaccines have obtained market approval since the emergence of SARS-CoV-2, uncertainties regarding the durability of the immune responses, adverse effects related to thrombosis, appearance of new variants, high production costs, dependence on a cold chain, and low availability of COVID-19 vaccines in developing countries warrant the development of novel vaccine technologies to curb the pandemic. The use of poxvirus MVA as a viral vector offers several advantages. It is a high-capacity vector for insertion of genes of interest and combines activation of humoral and cellular immunity with a good safety profile, even in children (25) and immunocompromised people (26). Moreover, MVA can be lyophilized, thereby eliminating the strict need for a cold chain during transport and storage of some currently available COVID-19 vaccines. In this regard, we have previously developed a vaccine candidate based on the poxvirus MVA vector expressing a human codon-optimized full-length SARS-CoV-2 S protein, termed MVA-S, which was highly immunogenic in mice, activating the production of robust levels of SARS-CoV-2-specific T-cell and humoral immune responses (10), and one or two doses of MVA-S protected 100% of transgenic K18-hACE2 mice from SARS-CoV-2 infection and mortality (10). Moreover, others have also recently described the generation of MVA-based vaccine candidates against SARS-CoV-2 that showed excellent immunogenicity and efficacy results in preclinical trials (10, 27–33), and two MVA-based vaccines against SARS-CoV-2 expressing either the S protein or S and N proteins are already in clinical trials (https://clinicaltrials.gov/ct2/show/NCT04895449 and https://clinicaltrials.gov/ct2/show/NCT04639466). The differences between our MVA-S vaccine candidate and that of other investigators are based on the use of human codon optimization of the S sequence, expression of the entire native S protein largely as a full-length size of 180 kDa, the thymidine kinase (TK) locus insertional site within the MVA genome, and nature of the synthetic early/late viral promoter. MVA is a non-replicative vector, with expression of the foreign genes limited to about 48 h after virus infection (34), but triggering immune responses similarly to a full replicating vaccinia virus vector (17). A recent study has shown that, when used as prime in a prime-boost regimen, an MVA vector expressing the SARS-CoV-2 S protein elicits similar levels of nAbs when compared to a replicative poxvirus vector (29). Furthermore, an MVA-based vaccine can be administered intranasally to improve mucosal immune responses, such as the presence of tissue-resident CD8 T cells and IgA in the lungs (27). Aerosol delivery of recombinant MVA has also been shown to be quite effective as a means of vaccination (35).

In the current study, we have tested the immunogenicity and protective efficacy of MVA-S in hamsters, revealing that one or two doses of MVA-S administered by the i.p. route was highly efficient to trigger binding IgG antibodies to SARS-CoV-2 S and RBD proteins, as well as high titers of nAbs against SARS-CoV-2. Cumulative studies on the evaluation of markers correlating with SARS-CoV-2 vaccine efficacy have pointed out that the induction of binding and nAbs correlates with protection (36). Even the one-dose MVA-S regimen was quite effective at inducing robust levels of binding and nAbs, being both further boostered by the second MVA-S vaccine dose. An important question is whether emerging VoC evades vaccine-induced immunity. We show here that the MVA-S vaccine candidate was quite effective in the capacity to neutralize a several VoCs, particularly alpha, gamma, and delta variants, with the beta variant requiring higher levels of antibodies to achieve a similar neutralization titer as for the other variants, findings also seen by others (27, 29). In another study, reduced neutralization of the beta variant did not result in reduced protection against beta challenge (37).

The Syrian hamster is a commonly used animal model for the study of SARS-CoV-2 infection (13, 15) and vaccine assessment (21, 27–29, 31, 37–39) because of similarities with humans regarding viral kinetics, antibody response, and COVID-19 clinical disease signs. In this study, animals immunized with MVA-S and challenged with live SARS-CoV-2 showed a marked reduction in virus infection and on lung pathology. Of importance, virus titers in the lungs exhibited up to 10^5^-fold reduction at the peak (4 dpi) compared to the MVA-WT control-infected animals, and this effect was observed even in animals receiving one dose of the vaccine. In this regard, a single dose of MVA-S was as efficient in reducing infectious viral titers in the lungs of hamsters as one dose of replication-competent poxvirus-vectored S protein assessed by others (29). By 14 dpi, there was no infectious virus remaining in the lungs. Histopathological scores of lung lesions were strongly reduced by MVA-S vaccination, even after one dose. A qualitative evaluation scoring different classes of injuries, like perivascular inflammation, peribronchiolar inflammation, and perivascular cuffs, showed marked reduction by vaccination.

Although our MVA-S vaccine candidate was able to control SARS-CoV-2 replication and pathology after challenge, both control and MVA-S-vaccinated hamsters displayed some loss in body weight during the first few days after challenge prior to full recovery by day 4. Notably, animals vaccinated by others using their MVA-based vaccines intranasally (27) or by scarification (29) showed comparable weight loss and recovery as seen in our study. In these studies, only intramuscularly vaccinated hamsters did not lose body weight after challenge and immunization resulted in complete absence of infectious virus in the lungs (29), although nAb titers were similar to those observed by us. Hamsters that were vaccinated intranasally with MVA-S showed no lung pathology nor infectious virus in the lungs after challenge, though the challenge virus inoculum was 20-fold lower than that used by us, leading to more than 10^2^-fold lower infectious titers in the lungs of control animals (27). Hence, obviously, the route of administration of the respective MVA-vectored vaccine, i.p. in our case, could influence outcome, next to other differences in the respective setup of animal experiments. Overall, consistent vaccine efficacy by the three different MVA-based candidates validates the use of the poxvirus vaccine platform for COVID-19.

While SARS-CoV-2 vaccines, particularly those of mRNA and adenovirus vectors, have been consolidated as part of the worldwide programs of vaccination of the population, there have been concerns on adverse effects observed in vaccinated individuals receiving the adenovirus vaccines and suffering thrombosis (40). The mechanism related to thrombosis has recently been demonstrated, fundamentally explained through electrostatic binding between the capsid of the adenovirus vector and platelet factor 4 (PF4) (41). This mechanism is unlikely to occur in MVA, as the poxviruses have different virion structural requirements, as well as encoding serine protease inhibitors (serpins) preventing thrombotic and thrombolytic pathways (42).

Overall, the robust immunogenicity and efficacy of the MVA-S vaccine candidate in hamsters support its further use as a vaccine against SARS-CoV-2 in clinical trials, alone or in combination with other vaccines.

## Data Availability Statement

The original contributions presented in the study are included in the article/**Supplementary Material**. Further inquiries can be directed to the corresponding authors.

## Ethics Statement

The ethical committee of KU Leuven (Belgium) approved housing conditions and experimental procedures (license p015/2020) according to institutional guidelines approved by the Federation of European Laboratory Animal Science Associations (FELASA).

## Author Contributions

Conceptualization: JG-A, KD, and ME. Formal analysis: RB and JG-A. Funding acquisition: JG-A, LC, JN, and ME. Investigation: RB, JG-A, PP, AL-F, DVL, TV, HJT, BW, and DA. Methodology: RB, JG-A, PP, AL-F, DVL, TV, HJT, BW, and DA. Resources: DM, EP, and ER. Supervision: RB, LC, JN, JG-A, KD, and ME. Validation: RB and JG-A. Visualization: RB and JG-A. Writing—original draft: RB, JG-A, KD, and ME. Writing—review and editing: all authors. All authors have read and agreed to the published version of the article.

## Funding

The authors declare that this study received funding from Fondo COVID-19 grant COV20/00151 [Spanish Health Ministry, Instituto de Salud Carlos III (ISCIII)], Fondo Supera COVID-19 grant (Crue Universidades-Banco Santander) and Spanish Research Council (CSIC) grant 202120E079 (to JG-A), CSIC grant 2020E84, La CaixaImpulse grant CF01-00008, Ferrovial and MAPFRE donations (to ME), a Spanish Ministry of Science and Innovation (MCIN)/Spanish Research Agency (AEI)/10.13039/501100011033 grant (PID2020-114481RB-I00 to JGA and ME), and internal funding from KU Leuven. This research work was also funded by the European Commission-NextGeneration EU through CSIC’s Global Health Platform (PTI Salud Global) (to JG-A and ME). The funders were not involved in the study design, collection, analysis, interpretation of data, the writing of this article or the decision to submit it for publication.

## Conflict of Interest

Authors DM, EP and ER were employed by the company Biofabri.

The remaining authors declare that the research was conducted in the absence of any commercial or financial relationships that could be construed as a potential conflict of interest.

## Publisher’s Note

All claims expressed in this article are solely those of the authors and do not necessarily represent those of their affiliated organizations, or those of the publisher, the editors and the reviewers. Any product that may be evaluated in this article, or claim that may be made by its manufacturer, is not guaranteed or endorsed by the publisher.
