# Supplementary material for: MVA-CoV2-S Vaccine Candidate Neutralizes Distinct Variants of Concern and Protects Against SARS-CoV-2 Infection in Hamsters
Source: Front Immunol. 2022 Mar 16;13:845969. doi: 10.3389/fimmu.2022.845969 (PMC8966703; doi:10.3389/fimmu.2022.845969)
Supplement: Supplementary file 1 [file DataSheet_1.pdf]

Supplementary information

MVA-CoV2-S Vaccine Candidate Neutralizes Distinct Variants of Concern and Protects Against SARS-CoV-2 Infection in Hamsters

Robbert Boudewijns [1], Patricia Pérez [2,3], Adrián Lázaro-Frías [2,3], Dominique Van Looveren [4], Thomas Vercruysse [4], Hendrik Jan Thibaut [4], Birgit Weynand [5], Lotte Coelmont [1], Johan Neyts [1], David Astorgano [2], Dolores Montenegro [6], Eugenia Puentes [6], Esteban Rodríguez [6], Kai Dallmeier [1,*], Mariano Esteban [2,*] & Juan García-Arriaza [2,3,*]

[1] KU Leuven Department of Microbiology, Immunology and Transplantation, Rega Institute, Laboratory of Virology and Chemotherapy, Leuven, Belgium. [2] Department of Molecular and Cellular Biology, Centro Nacional de Biotecnología (CNB), Consejo Superior de Investigaciones Científicas (CSIC), Madrid, Spain. [3] Centro de Investigación Biomédica en Red de Enfermedades Infecciosas (CIBERINFEC), Madrid, Spain. [4] KU Leuven Department of Microbiology, Immunology and Transplantation, Rega Institute, Translational Platform Virology and Chemotherapy (TPVC), KU Leuven, Leuven, Belgium. [5] KU Leuven Department of Imaging and Pathology, Translational Cell and Tissue Research, KU Leuven, Leuven, Belgium. [6] Biofabri, O Porriño, Pontevedra, Spain.

* Correspondence: Juan García-Arriaza (jfgarcia@cnb.csic.es), Mariano Esteban (mesteban@cnb.csic.es) and Kai Dallmeier (kai.dallmeier@kuleuven.be)

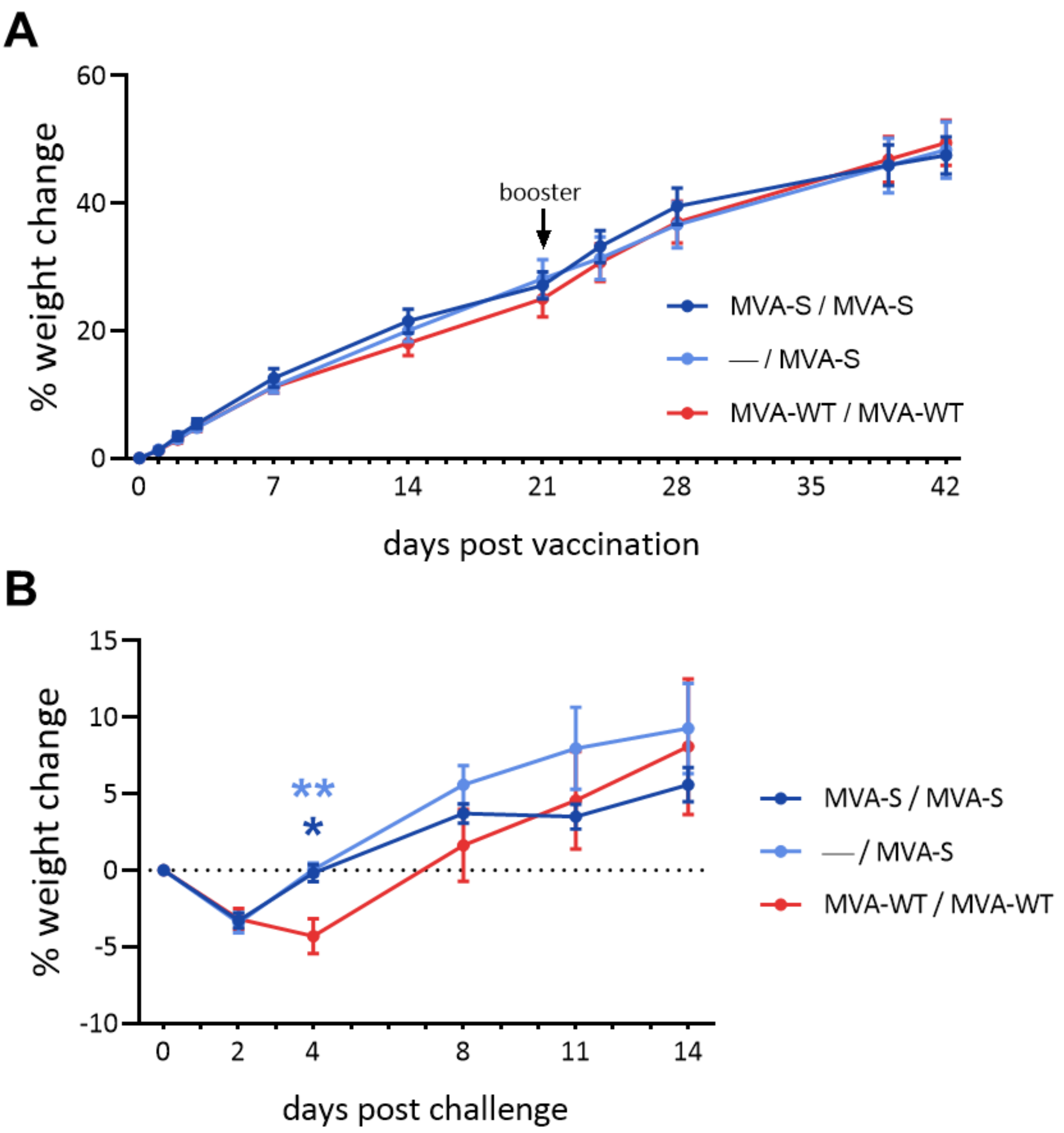

Supplementary Figure 1. Hamster body weight change throughout the course of the experiment. Data are expressed as means ± SEM of percentage body weight change compared to the (A) beginning of the experiment (day 0) or (B) day of challenge (d42). Statistical significance between groups was calculated by Mann-Whitney test (* $p<0.05$, ** $p<0.01$).

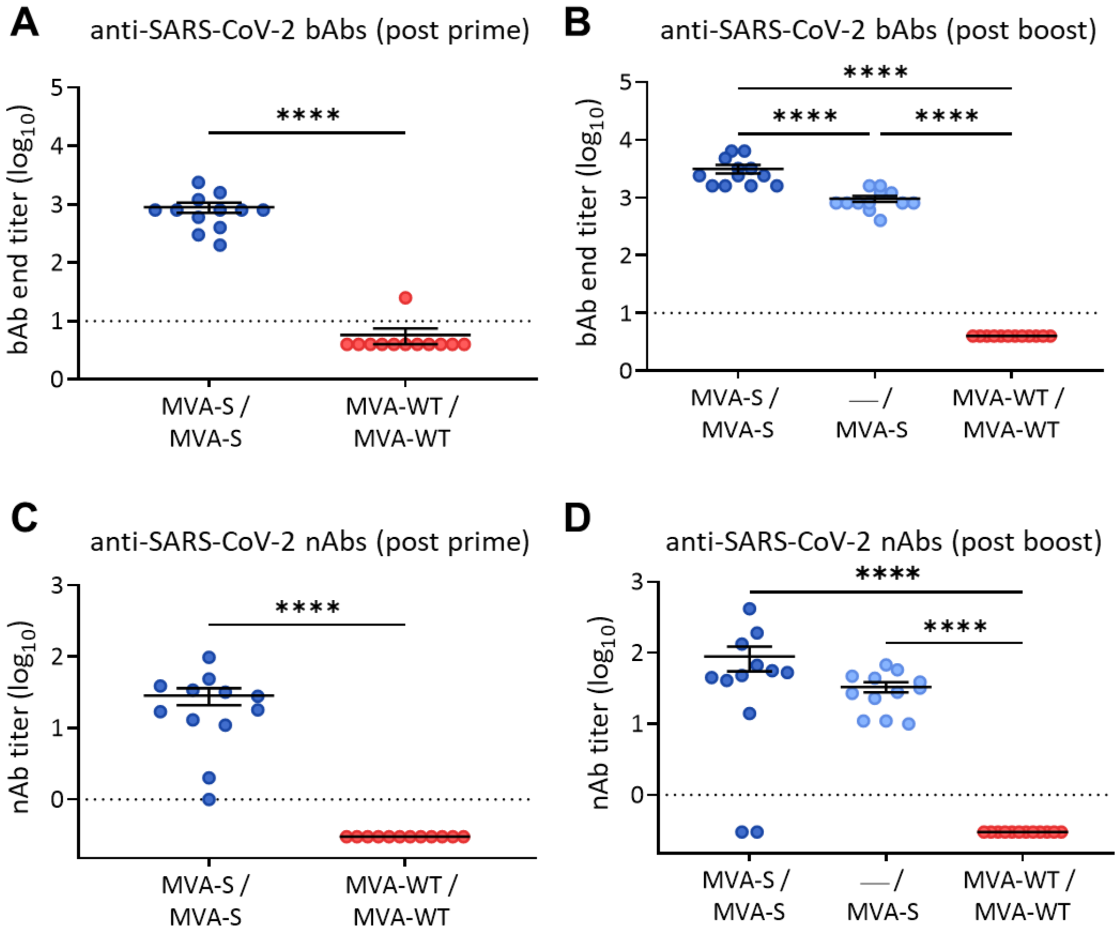

Supplementary Figure 2. SARS-CoV-2-specific humoral immune responses induced by MVA-S vaccination in hamsters. (A, B) Total binding IgG antibody titers determined by IIFA in serum from vaccinated hamsters collected on (A) day 21 (post prime but prior to boost), and (B) day 39 (18 days postboost, and prior to challenge). **(C, D)** Neutralizing antibody titers measured by SNT in hamster serum collected on (C) day 21 and (D) day 39. Dotted lines represent the limit of detection. Data presented as means ± SEM. Statistical significance between groups was calculated by Mann-Whitney test (**** $p<0.0001$).
